# Thermoresponsive sol–gel containing probiotic’s cell free supernatant for dental caries prophylaxis

**DOI:** 10.1080/20002297.2021.2012390

**Published:** 2021-12-19

**Authors:** Panithi Raknam, Neelam Balekar, Rawee Teanpaisan, Thanaporn Amnuaikit

**Affiliations:** a Department of Pharmaceutical Technology and Drug Delivery System Excellence Center, Faculty of Pharmaceutical Sciences, Prince of Songkla University, Songkhla, Thailand; b IPS Academy College of Pharmacy, Indore, India; c Department of Stomatology and the Common Oral Diseases and Epidemiology Research, Faculty of Dentistry, Prince of Songkla University, Songkhla, Thailand

**Keywords:** Cell free supernatant, *Lactobacillus rhamnosus* SD11, poloxamer 407, thermoresponsive polymer, dental caries

## Abstract

**Background:**

*Lactobacillus rhamnosus* SD11 is a probiotic derived from the human oral cavity and has potential being used for dental prophylaxis. The cell free supernatant (CFS) of *L. rhamnosus* SD11 has good antimicrobial and antioxidant effects.

**Aim:**

This study aimed to incorporate CFS of the probiotic into thermoresponsive copolymers to create a sol–gel formulation.

**Methods:**

The sol–gel formulation was developed using Poloxamer 407 as the main polymer, which was mixed with natural polymers such as gellan gum, sodium alginate, and xyloglucan in different proportions. The sol–gel formulations were characterized based on their physicochemical parameters such as appearance, pH, viscosity, flow-ability in low temperature, antioxidant and antibacterial activity. An *in vitro* release study was performed using Franz’s diffusion cell and the stability was determined under freeze-thaw cycle conditions.

**Results:**

The combination of 15% w/v of poloxamer 407 with 0.5% w/v of sodium alginate was the best sol–gel formulation to deliver the CFS of the probiotic.

**Conclusion:**

This study was successful in creating a sol–gel formulation using a thermoresponsive copolymer, that could efficiently deliver CFS of the probiotic *L. rhamnosus* SD11.

**Figure:**
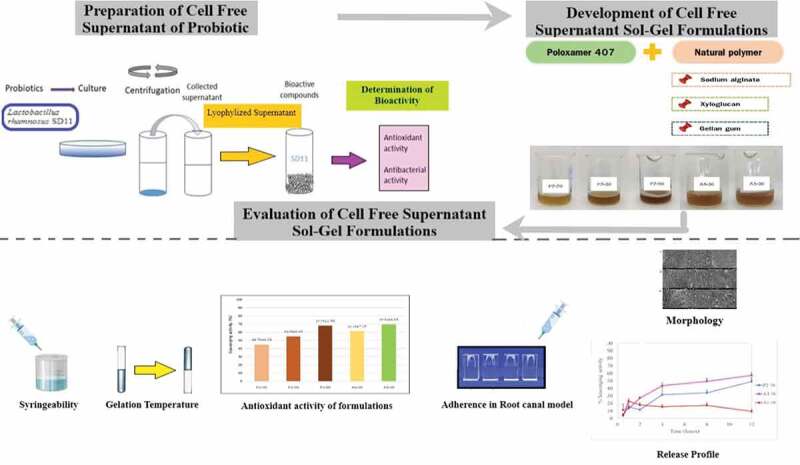

## Introduction

*Lactobacillus rhamnosus* SD11 is a probiotic that is derived from the human oral cavity. It is a facultative anaerobic, Gram-positive rod-shaped bacterium, which is included in the *Lactobacillus* genus [1]. This probiotic strain has become popular in pharmaceutical and food industries recently, because of its ability to produce bacteriocins and bio-active compounds. Previous studies found that the cell free supernatant (CFS) of *L. rhamnosus* SD11 contains various bio-active compounds, especially bacteriocins which are beneficial for dental caries prophylaxis [2]. The CFS of *L. rhamnosus* SD11 also contains fermencin SD11 (MW 33,593.4 Da), which is a broad spectrum antimicrobial bacteriocin active against Gram positive and negative bacteria, as well as yeast. The antimicrobial activity of fermencin SD11 is stable within pH 3.0 to 7.0, and at temperatures between 60°C and 80°C [3,4]. Results from *in vitro* and *in vivo* studies have shown that *L. rhamnosus* SD11 has a good potential in dental caries prophylaxis and is safe for both short term as well as long-term use [3,4]. Also, a clinical study of *L. rhamnosus* SD11 provided evidence of its effect in preventing dental carries [3–5].

CFS of probiotics is usually considered a waste in many pharmaceutical and food industries, and is rarely or never used for creating dental products. Therefore, this study aimed to utilize CFS of *L. rhamnosus* SD11 as a new active ingredient in dentistry by developing a sol–gel formulation to deliver it. The CFS of *L. rhamnosus* SD11 could be used in the oral cavity to reduce bacteria which cause tooth decay, if administered in adequate amounts [6], with a prolonged contact time at the infected area.

Various oral cavity formulations such as mouthwashes, gels, pastes, ointments, etc. are used for the treatment of periodontitis. However, these formulations cannot deliver the active ingredient deeply into the root canal or the infected area with high enough concentrations and long contact times. Thermoresponsive polymer was one of the most suitable materials for developing a formulation that aligned with the purpose of this study. The sol–gel formulation developed with thermoresponsive copolymers is an aqueous solution injectable by syringe into the oral cavity at low temperatures, and inside the oral cavity it transforms to a gel upon reaching body temperature, thereby releasing bio-compounds for tooth decay prevention.

**Figure 1.**
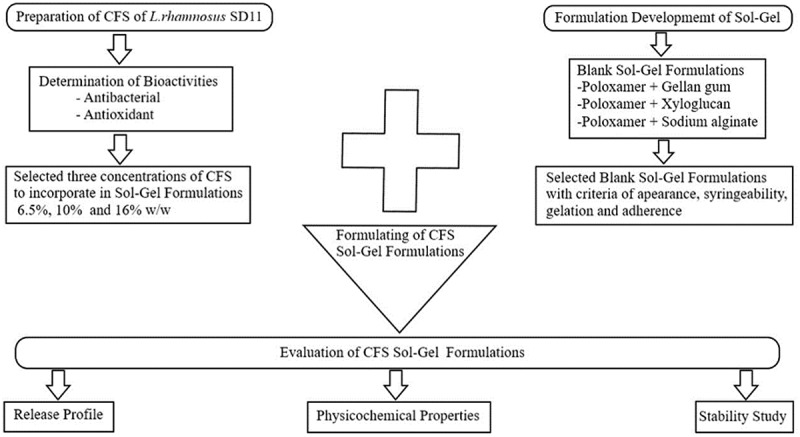
A flow chart of development and evaluation of CFS sol–gel formulations

Thermoresponsive polymers are materials that respond to a change in temperature. They are used for biomedical applications including drug delivery, tissue engineering and gene delivery [7]. Poloxamer 407 is a thermoresponsive polymer, a family of triblock copolymers with two hydrophilic blocks of polyethylene oxide and one hydrophobic block of polypropylene oxide [8]. Poloxamer 407 was of special interest in this study on drug delivery because, it is a thermoreversible polymer that is an aqueous solution at low temperatures, but once it is exposed to temperatures in the range of the human body, it goes through the process of gelation to form a semisolid gel [8]. Moreover, a previous study have shown that the use of poloxamer 407 gels for the delivery of lidocaine extended the contact time of the drug at the site of injection, and sustained the release of the drug [9]. Therefore, sol–gel injectable formulations that consist of poloxamer 407 is a good candidate for delivery of CFS of *L. rhamnosus* SD11. However, only utilizing poloxamer 407 would not be sufficient to provide an adequate adherence property to the sol-gel formulation in the root canal. Therefore, natural polymers such as gellan gum, sodium alginate and xyloglucan were also used to develop formulations together with poloxamer 407 in various ratios of concentrations. Gellan gum is a water-soluble anionic polysaccharide which grows naturally on water lilies, but it can also be artificially produced by fermenting sugar with the bacterium *Sphingomonas elodea* [10]. Sodium alginate is the sodium salt of alginic acid, whereas alginates are derived from brown seaweeds which belong to the class *Phaeophyceae* [11]. Xyloglucan is a non-ionic, neutral, branched polysaccharide consisting of a cellulose-like backbone that carries xylose and galactosyl-xylose substituents. It is a hemicellulose extracted from the seed of the tamarind tree [12]. Physicochemical properties of formulations such as antioxidant and antimicrobial activity, injectability at low temperatures, adherence, release of CFS of a probiotic from the gel, and stability of the formulations were also investigated in this study. The flow chart of the development and evaluation of the sol-gel formulations is shown in Figure 1.

### Materials and methods

Sodium alginate, xyloglucan, methylparaben and propylparaben were obtained from P.C. Drug Center Co., Ltd. (Thailand). Poloxamer 407 was received from BASF Chemcat Thailand Limited. Gellan gum was purchased from Kanto chemical CO., INC (Japan). Ascorbic acid was obtained from Sigma-Aldrich (China). 2,2-Diphenyl-1-picrylhydrazyl (DPPH) was purchased from Sigma-Aldrich (Germany). The solvents and other analytical chemical reagents were bought from Labscan Asia Co., Ltd. (Bangkok, Thailand).

### Preparation and lyophilization of CFS from *L. rhamnosus* SD11

*L.rhamnosus* SD11 (human oral origin) strains were obtained from the previous study of Piwat etal. [13]. The strains had already been identified and they were stored at −80°C at the Department of Stomatology, Faculty of Dentistry, Prince of Songkla University, Thailand. They were cultured on de Man Rogosa Sharpe agar (MRS) at 37°C for 24 h under anaerobic conditions. A single colony of *L.rhamnosus* SD11 was cultured in MRS broth and incubated anaerobically for 24 h. The cultures were centrifuged at 8,000 rpm for 10 min to remove bacterial cells. The obtained CFS was frozen overnight at −80°C prior to freeze-drying in a vacuum freeze dryer (Scanvac Cool Safe^TM^, Denmark) for 48 h at −110°C. The lyophilized samples were kept at −20°C until further use [14].

### Pathogens and growth conditions

*Staphylococcus aureus* ATCC 29213 and *Streptococcus mutans* ATCC 25178 were cultured on blood agar (DifcoTM, USA) which was supplemented with 5% v/v blood. The strains were incubated anaerobically at 37°C for 24 h.

### Bioactivity study of CFS of *L. rhamnosus*SD11

Bioactivity of CFS of *L.rhamnosus* SD11 was determined in terms of antibacterial and antioxidant effects. The minimal inhibitory concentration (MIC) and minimal bactericidal concentration (MBC) were investigated by broth microdilution assay for determining the antibacterial activity [14]. Briefly, 100 µl of CFS with brain heart infusion (BHI) broth (DifcoTM, USA) was seeded in a 96 wells plate to obtain final concentrations ranging from 0.04 to 10 mg/ml. One hundred microliter of each pathogen (10^8^ CFU/ml) was added into each well and incubated at 37°C under appropriate conditions. The supernatant without pathogens was used for sterility control. The pathogen suspensions were used as positive control, and the BHI broth as negative control. DPPH radical scavenging activity assay was used to evaluate antioxidant activity [15]. DPPH 70% v/v ethanolic solution was freshly prepared before use and each sample was diluted to 1:5 in 50% v/v ethanol, then centrifuged at 12,000 g for 25 min at 25°C. Next, 100 µl of each supernatant was added to 100 µl of the DPPH solution and mixed thoroughly, after which the solutions were kept in the dark for 30 min for the reaction to occur. The absorbance was measured at 517 nm by a microplate reader (SPECTRO star Nano, BMG LABTECH Gmbh, Germany). Calibration was performed with L-ascorbic acid as a standard.

### Preparation of the thermoresponsive sol-gel formulation

**Figure 2.**
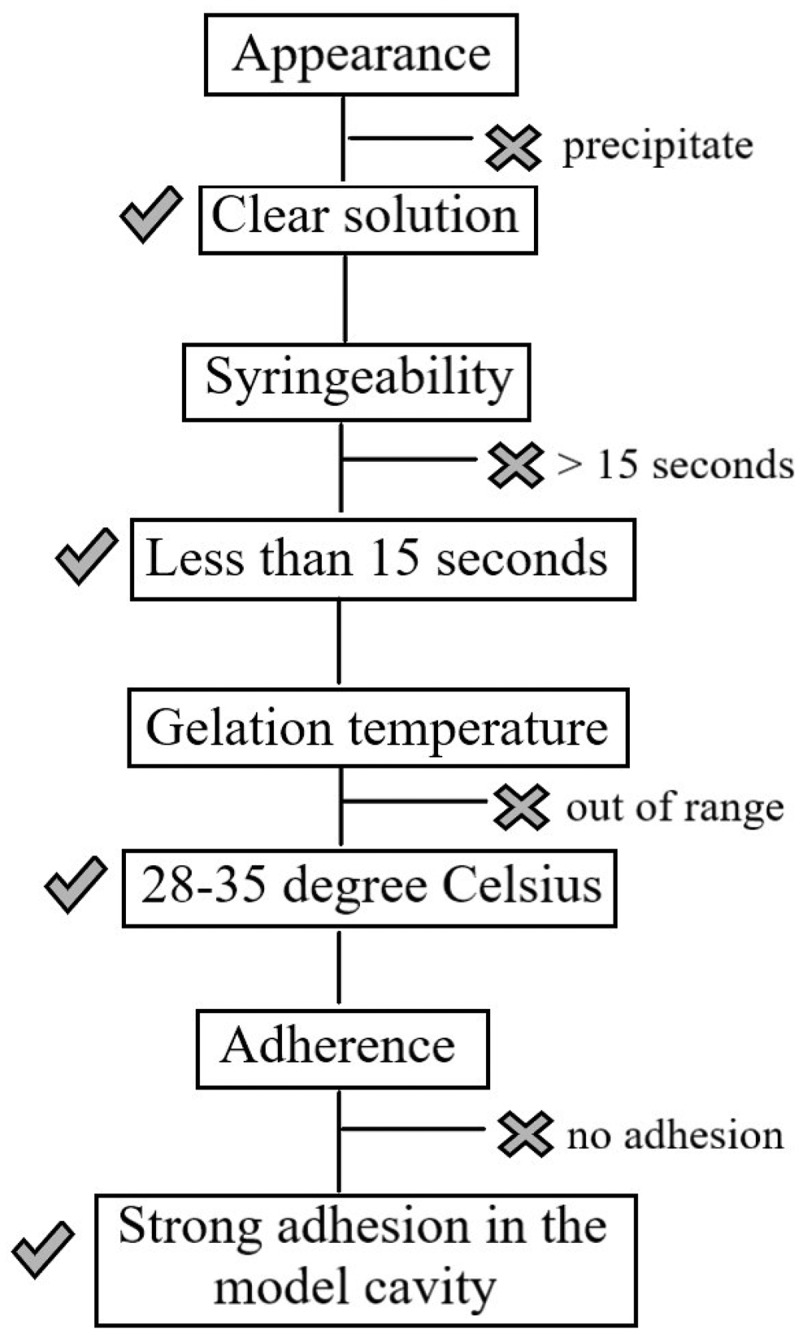
A tree diagram of properties criteria for selection of blank copolymer solutions

Thermoreversible gelation is a characteristic of Poloxamer 407 which gives it the ability to remain as a solution at low temperatures (4°C to 24°C), and as a semisolid gel at high temperatures (25°C and above). Poloxamer 407 and natural polymers were combined in various ratios and concentrations as shown in Table 1. The solution containing only poloxamer 407 was prepared by dispersing poloxamer 407 in cool water at 4°C and storing it in a refrigerator until a clear solution was observed [16]. The combination of poloxamer 407 and xyloglucan solution was prepared as follows: xyloglucan was dispersed in hot water at 50°C using a magnetic hotplate stirrer for 4 h, after which poloxamer 407 was dispersed in this solution at 4°C. The solution was then kept in a refrigerator until a clear solution was obtained [16]. Other solutions where poloxamer 407 was combined with gellan gum and sodium alginate respectively were also prepared in a similar proces, but the temperature of the hot water was 95°C [17] in case of gellan gum, and room temperature (25°C) [14] in case of sodium alginate. Three concentrations (6.5, 10 and 16% w/w) of CFS of *L.rhamnosus* SD11 were added to the formulations that were selected based on the desirable criteria (Figure 2). The compositions are displayed in Table 2. These formulations were taken in a beaker and continuously stirred until a uniform solution was obtained (4°C). Thereafter, to adjust the pH of these formulations to approximately 7, 0.8% w/w of triethanolamine was added to formulations containing 6.5 and 10% w/w of CFS, and 1% w/w of triethanolamine was added to the formulations containing 16% w/w of CFS, respectively.

**Table 1.** The composition of blank copolymer solutions

Ingredients (%w/w)	P1	P2	P3			
Poloxamer 407	15	16	17			
Methylparaben	0.1	0.1	0.1			
Propylparaben	0.02	0.02	0.02			
Water	84.88	83.88	82.88			
**Ingredients (%w/w)**	**X1**	**X2**	**X3**	**X4**	**X5**	**X6**
Poloxamer 407	15	16	17	15	16	17
Xyloglucan	0.1	0.1	0.1	1	1	1
Methylparaben	0.1	0.1	0.1	0.1	0.1	0.1
Propylparaben	0.02	0.02	0.02	0.02	0.02	0.02
Water	84.78	83.78	82.78	83.88	82.88	81.88
**Ingredients (%w/w)**	**G1**	**G2**	**G3**	**G4**	**G5**	**G6**
Poloxamer 407	15	16	17	15	16	17
Gellan gum	0.3	0.3	0.3	0.6	0.6	0.6
Methylparaben	0.1	0.1	0.1	0.1	0.1	0.1
Propylparaben	0.02	0.02	0.02	0.02	0.02	0.02
Water	84.58	83.58	82.58	84.28	83.28	82.28
I**ngredients (%w/w)**	**A1**	**A2**	**A3**	**A4**	**A5**	**A6**
Poloxamer 407	15	16	17	15	16	17
Sodium alginate	0.1	0.1	0.1	0.5	0.5	0.5
Methylparaben	0.1	0.1	0.1	0.1	0.1	0.1
Propylparaben	0.02	0.02	0.02	0.02	0.02	0.02
Water	84.78	83.78	82.78	84.38	83.38	82.38

**Table 2.** The composition of the selected CFS of *L. rhamnosus* SD11 sol–gel formulations

Rx	CFS	Poloxamer 407	Xyloglucan	Sodium alginate	Methyl paraben	Propyl paraben	Triethano lamine	Water
P1-20	6.5	15.0	-	-	0.1	0.02	0.8	77.58
P1-30	10	15.0	-	-	0.1	0.02	0.8	74.08
P1-50	16	15.0	-	-	0.1	0.02	1	67.88
P2-20	6.5	16.0	-	-	0.1	0.02	0.8	76.58
P2-30	10	16.0	-	-	0.1	0.02	0.8	73.08
P2-50	16	16.0	-	-	0.1	0.02	1	66.88
X1-20	6.5	15.0	0.1	-	0.1	0.02	0.8	77.48
X1-30	10	15.0	0.1	-	0.1	0.02	0.8	73.98
X1-50	16	15.0	0.1	-	0.1	0.02	1	67.78
X2-20	6.5	16.0	0.1	-	0.1	0.02	0.8	76.48
X2-30	10	16.0	0.1	-	0.1	0.02	0.8	72.98
X2-50	16	16.0	0.1	-	0.1	0.02	1	66.78
A4-20	6.5	15.0	-	0.5	0.1	0.02	0.8	77.08
A4-30	10	15.0	-	0.5	0.1	0.02	0.8	73.58
A4-50	16	15.0	-	0.5	0.1	0.02	1	67.38
A5-20	6.5	16.0	-	0.5	0.1	0.02	0.8	76.08
A5-30	10	16.0	-	0.5	0.1	0.02	0.8	72.58
A5-50	16	16.0	-	0.5	0.1	0.02	1	66.38

### Determination of physicochemical properties of the sol-gel

#### Physical properties characterization

All formulations were assessed for their color and appearance (clear or turbid) by visual assessment. The pH of each formulation was measured at 25 ± 1°C using a digital pH meter, and the test was done in triplicate. The viscosity of the formulations was measured at 25°C using Brookfield Programmable Rheometer, Model RVT, spindle-type SC4-31 at 100 rpm, and spindle-type T-bar at 5 rpm under controlled temperatures by the compact low-temperature thermostat Model 6 at 5°C, 25°C, and 37°C [15]. Furthermore, the rheological properties of the gels finally obtained (37°C) were determined using a Rheometer model DV-III (Brookfield DV III Ultra Programmable Rheometer, USA) with T-F spindle. The measurement was performed at five different shearing speeds of 5, 10, 15, 20 and 25 rpm. The mechanical properties of gel formulations were evaluated using a Texture Analyzer (TA-TX plus, Surrey, UK) with P-0.5 R probe and a single press mode with a pre-test speed of 1 mm/s. The probe was a punctured into each sample at a constant speed (2 mm/s) at a depth of 10 mm, after which it was returned to the starting point with a post-test speed of 2 mm/s. The Texture Analyzer was able to reveal the mechanical properties of the gels such as gel strength, rupture strength, brittleness and adhesiveness. The morphology of selected CFS formulations were determined by a Scanning Electron Microscope (SEM) (SU3900, Hitachi, Japan) at an accelerating voltage of 20 kV. The samples were dried using a Freeze Dryer (FTS Systems DuraDry, USA) and coated with gold in a sputter coater (SPI 11430/11,428, USA). Samples were observed at 200 x, 1,000 x and 5,000 x magnification.

#### Syringeability and injectability evaluation

A 5 ml syringe was used to take 1 ml of the polymer solution at temperatures of 5°C and 25°C, which was controlled by an E100 Ecoline Star Edition Circulating Water Bath Immersion Heater. A timer was used to measure the time taken by the polymer to fully enter and fully exit the syringe [18]. The criterion was that, the total time taken was not to be over 15 sec.

#### Gelation temperature evaluation

Two grams of polymer solution was taken in a test tube and kept under controlled temperatures in the range 20–40°C. The temperature was increased in steps of 1°C at a time, and during each step, the solution was observed for signs of gelation (no flow when the test tube was held upside down), and finally the temperature at which there was no flow upside down was recorded as the gelation temperature [18].

#### Evaluation of root canal adherence

**Figure 3.**
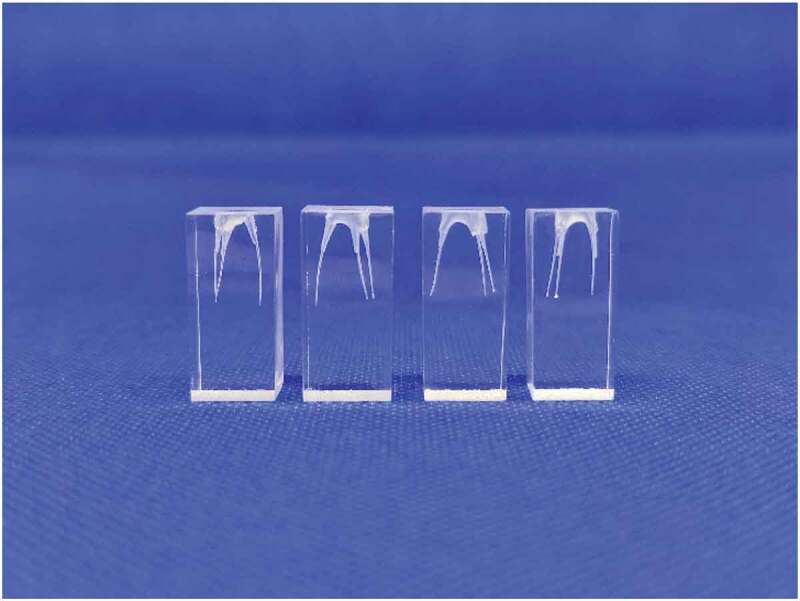
The root canal dental model

Evaluation of root canal adherence was performed by taking 0.5 ml of polymer solution in a syringe and filling it in a root canal model (Figure 3), under a controlled temperature of 37°C. After 5 min, the dental model was held upside down to observe whether the formulation could stay in the root canal firmly, or if it tended to flow out. The formulation was deemed to have good adherence property if it stayed inside the root canal firmly, without any tendency to flow out when held upside down.

#### Antioxidant activity determination of the formulation

DPPH radical scavenging activity assay was used to evaluate the antioxidant activity of the formulation [15]. One gram of formulation was homogeneously mixed in 5 ml of water using a magnetic stirrer, after which it was diluted with water to 100 ml. Next,100 µl of this solution was added to 100 µl of the DPPH solution, and the antioxidant activity was determined using the same method mentioned previously in the bioactivity study of CFS.

#### Antibacterial activity determination of the formulation

Antibacterial activities of selected sol–gel formulations (P2-50, A4-30 and A5-30) were evaluated by an agar plate diffusion assay. One millilitre of *S.aureus* (10^8^ CFU/ml) was mixed with 20 ml of melted BHI (Difco TM, USA). The mixture was poured into a plate containing metal cups (6 mm diameter). The metals cups were removed as soon as the agar was solidified. Sol–gel formulations were loaded into the well at full volume capacity by using a syringe. Blank sol-gel was used as control. The plate was then incubated anaerobically (80% N_2_,10% H_2_, and 10% CO_2_) at 37°C for 24 h. The antibacterial activity was evaluated by observing the presence of an inhibition zone. Each experiment was done in triplicate [19].

### Release study of the sol–gel formulation containing CFS of *L. rhamnosus* SD11

The release of CFS from the formulation was studied using a modified Franz-diffusion cell (Hanson 57–6 M, USA). The receptor fluid in the receptor compartment was 12 ml of phosphate buffer solution (pH 7.4) which was continuously stirred at 300 rpm with a magnetic stirrer. The circulating water bath was set at 37 ± 1°C.A hydrated cellulose acetate membrane with a Molecular Weight Cut Off (MWCO) of 3,500 Dalton was placed between donor and receptor compartments. One gram of each formulation was put on to the donor compartment with an effective diffusion area of 1.77 cm^2^. At 0.5, 1, 2, 4, 6 and 12 h, the receptor fluid was withdrawn and immediately replaced with an equal volume of the thermoregulated (37°C) fresh receptor fluid. The release of active components from the formulations was evaluated by determining the antioxidant activity of the withdrawn samples at various time intervals, using the DPPH assay [14].

### Stability study of the sol–gel formulations containing CFS of *L. rhamnosus* SD11

Stability of the selected sol–gel formulations was evaluated by the freeze thaw (FT) cycle method. The selected formulations were kept in a refrigerator at a temperature of 4 ± 2°C for 24 h, after which they were kept at 45 ± 2°C in a hot air oven for 24 h. This process was considered as one cycle of the freeze-thaw method. The physical properties including colour, texture, pH and viscosity as well as antioxidant activity were observed before and after five freeze-thaw cycles. All the experiments were done in triplicate [15].

### Data analysis

Results are expressed as mean ± standard deviation (SD). The data were analysed with either one-way analysis of variance (ANOVA) or student t-test. *P* < 0.05 was considered statistically significant.

## Results

### Bioactivity of CFS of *L. rhamnosus* SD11

CFS of *L. rhamnosus* SD11 showed antibacterial activity against *S. mutans* and *S. aureus* with MIC 9.38 ± 0.0 and 12.5 ± 0.0 mg/ml and MBC 18.8 ± 0.0 and 25.0 ± 0.0 mg/ml, respectively. The antioxidant activity of CFS of *L. rhamnosus* SD11 was evaluated by the DPPH radical scavenging assay. Ascorbic acid was used as standard. EC_50_ values were calculated from linear plots and the results were summarized. In DPPH scavenging assay, low EC_50_ value indicates potent antioxidant activity. EC_50_ values of CFS of *L. rhamnosus* SD11 was 3.19 ± 0.03 mg/ml and ascorbic acid was 6.32 ± 0.02 µg/ml.

### Physicochemical properties of the sol–gel formulations

**Figure 4.**
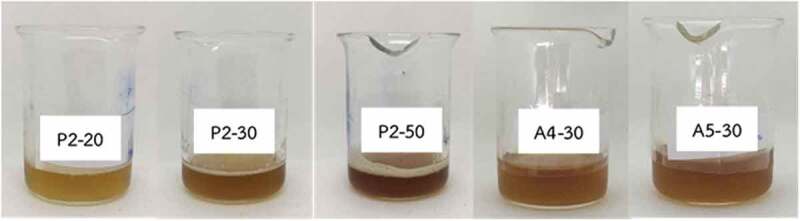
Appearance of the selected CFS of *L. rhamnosus* SD11 sol–gel formulations. P2-20, P2-30 and P2-50 composed of poloxamer 407(16% w/w) and CFS, but the concentration of CFS was different in these formulations which was 6.5, 10 and 16% w/w respectively. A4-30 and A5-30 contained CFS (10% w/w) and sodium alginate (0.5% w/w), but they had different concentrations of poloxamer 407 which were 15 and 16% w/w respectively

**Figure 5.**
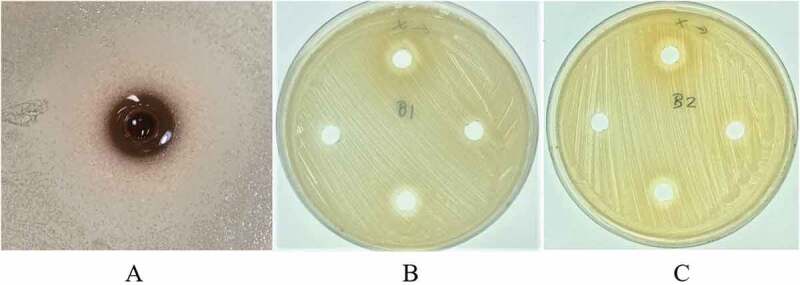
The inhibition zone of CFS of *L. rhamnosus* SD11 sol–gel formulation A4-30 (**A**), and blank sol–gel formulations P2 (**B**) and A4 (**C**)

Appearance and pH of the blank copolymer solution is shown in Table 3. Based on physical appearance, formulations which were composed of xyloglucan 1% w/w (X4-X6) were taken out due to precipitation. pH of all formulations was in the range 6.84–7.04. In case of syringeability and injectability of all formulations, most of them could be easily taken into the syringe and expelled from the syringe in less than 15 s, except the formulations containing poloxamer and gellan gum. All different ratios of formulation containing poloxamer mixed with gellan gum made the formulation viscous and resulted in a failure of syringeability and injectability determination. Therefore, the formulations G1-G6 were taken out from a list of potential candidates of blank copolymer solutions that were to be mixed with CFS of *L. rhamnosus* SD11. Gelation temperature and adherence of formulations are shown in Table 3. Interestingly, 15% w/w of poloxamer 407 mixed with 0.1% w/w (A1) and 0.5% w/w (A4) of sodium alginate gave higher gelation temperatures than all other formulations. All formulations were able to adhere well in the root canal model except A1, which flowed outside from the cavity at 37°C, when the model was held upside down. From the results of physical properties of blank copolymer solutions, six formulations (P1, P2, X1, X2, A4 and A5) were selected to incorporate CFS of *L. rhamnosus* SD11. Each formulation was prepared in ratios of twenty (6.5% w/w), thirty (10% w/w) and fifty (16% w/w) times the EC_50_ value (3.19 ± 0.03 mg/ml) of CFS of *L. rhamnosus* SD11 as shown in Table 2. The gelation temperature of all formulations containing CFS of *L. rhamnosus* SD11 is shown in Table 4. The criteria for selection of CFS of *L. rhamnosus* SD11 sol-gel formulations was gelation temperature in the range 25–35°C. Therefore, five formulations (P2-20, P2-30, P2-50, A4-30 and A5-30) were selected to evaluate other physicochemical properties. The appearance of these five CFS of *L. rhamnosus* SD11 sol-gel formulations is displayed in Figure 4. All the formulations were clear and brown in color, with the intensity of brown color increasing with increasing concentrations of CFS of *L. rhamnosus* SD11. pH, adherence, and antioxidant activity of CFS of *L. rhamnosus* SD11 sol-gel formulations are shown in Table 5. Three formulations containing CFS of *L. rhamnosus* SD11 (P2-50, A4-30 and A5-30) with high antioxidant activity were selected for the viscosity study at various temperatures (Table 6). The viscosity of the three selected formulations increased with an increase in temperature. Syringeability and injectability of three formulations containing CFS of *L. rhamnosus* SD11 were good at the temperature of 5°C, as all three formulations could easily be taken inside the syringe and expelled from the syringe in less than 15 s, since they were in a liquid state. However, at a temperature of 25°C, only formulation A4-30 displayed good syringeability and injectability, while the other formulations were viscous, and difficult to take into the syringe. Furthermore, three of the CFS of *L. rhamnosus* SD11 sol-gel formulations (P2-50, A4-30 and A5-30) showed inhibition zones following agar plate diffusion, while the blank sol-gel could present only a small clear zone (Table 7). The inhibition zone of A4-30 and blank sol-gel (A4) is displayed in Figure 5. Both of the blank sol-gel formulations (P2 and A4) containing preservative did not affect the inhibition zone on agar plates much, compared to the formulations containing CFS of *L. rhamnosus* SD11 (A4-30). Preservative was used in both the blank and CFS of *L. rhamnosus* SD11 sol-gel formulations to prevent products from microbial contamination and to prolong their shelf life, especially because of the high amount of water content in the formulations. The inhibition zone of the blank sol-gel formulation containing preservative showed that the preservative did not affect the antimicrobial ability, which in turn assures that the product containing CFS of *L. rhamnosus* SD11 will also not be affected by the preservative contained in the formulation.

**Table 3.** Appearance, pH, gelation temperature and adherence of formulations

Formulations	Appearance	pH	Gelation (°C)	Adherence
P1	Clear and colorless	6.88 ± 0.05	31.67 ± 0.58	✓
P2	Clear and colorless	6.97 ± 0.00	29.67 ± 0.58	✓
P3	Clear and colorless	7.01 ± 0.03	27.67 ± 0.58	✓
X1	Turbid and colorless	7.04 ± 0.06	31.33 ± 0.58	✓
X2	Turbid and colorless	7.04 ± 0.02	28.33±±0.58	✓
X3	Turbid and colorless	7.04 ± 0.01	24.33 ± 0.58	✓
X4	Brown color with precipitate	-	-	-
X5	Brown color with precipitate	-	-	-
X6	Brown color with precipitate	-	-	-
G1	Clear and colorless	6.99 ± 0.04	-	-
G2	Clear and colorless	6.97 ± 0.01	-	-
G3	Clear and colorless	6.98 ± 0.02	-	-
G4	Clear and colorless	7.01 ± 0.05	-	-
G5	Clear and colorless	7.00 ± 0.08	-	-
G6	Clear and colorless	7.03 ± 0.01	-	-
A1	Clear and colorless	6.96 ± 0.07	>40.00	x
A2	Clear and colorless	6.95 ± 0.04	27.67 ± 0.58	✓
A3 A4	Clear and colorless Clear and colorless	6.93 ± 0.02 6.84 ± 0.02	26.33 ± 0.58 32.33 ± 0.58	✓ ✓
A5	Clear and colorless	6.91 ± 0.05	29.33 ± 0.58	✓
A6	Clear and colorless	6.92 ± 0.00	24.67 ± 0.58	✓

**Table 4.** Gelation temperature of formulation containing CFS of *L. rhamnosus* SD11

Formulations	Gelation temperature (°C) (mean ± SD)	
	CFS of *L. rhamnosus* SD11	Blank copolymer
P1-20	>40	31.67 ± 0.58
P1-30	>40	
P1-50	>40	
P2-20	27.67 ± 0.58	29.67 ± 0.58
P2-30	29.33 ± 0.58	
P2-50	27.33 ± 0.58	
X1-20	>40	32.33 ± 0.58
X1-30	>40	
X1-50	24.67 ± 0.58	
X2-20	21.33 ± 0.58	29.33 ± 0.58
X2-30	23.33 ± 0.58	
X2-50	20.33 ± 0.58	
A4-20	22.33 ± 0.58	31.33 ± 0.58
A4-30	27.67 ± 0.58	
A4-50	21.33 ± 0.58	
A5-20	23.33 ± 0.58	28.33 ± 0.58
A5-30	25.33 ± 0.58	
A5-50	20.33 ± 0.58

**Table 5.** pH, adherence and antioxidant activity of CFS *L. rhamnosus* SD11 sol–gel formulations

Formulations	pH	adherence	Antioxidant activity (%)
P2-20	7.20 ± 0.02	✓	44.74 ± 6.24
P2-30	7.26 ± 0.02	✓	54.55 ± 6.45
P2-50	7.30 ± 0.02	✓	67.79 ± 1.98
A4-30	7.07 ± 0.02	✓	61.15 ± 7.19
A5-30	7.17 ± 0.03	✓	69.52 ± 4.63

**Table 6.** Viscosity values of the selected formulations containing CFS of *L. rhamnosus* SD11 at various temperatures

Temperature	Viscosity (cPs)		
	P2-50	A4-30	A5-30
5	103.4433 ± 6.50	94.48 ± 0.60	99.18 ± 4.45
25°C	24000 ± 200.00	431.57 ± 30.13	15,733.33 ± 152.75
37°C	36966.67 ± 208.16	20,900 ± 300	32,233.33 ± 152.75

**Table 7.** Inhibition zones of formulations on *Staphylococcus aureus* agar plates

Formulations	Inhibition zone (Average (n = 3), ± SD., mm
Blank sol-gel	6.00 ± 0.00
P2-50	17.05 ± 0.82
A4-30	16.48 ± 0.41
A5-30	15.28 ± 0.34

**Figure 6.**
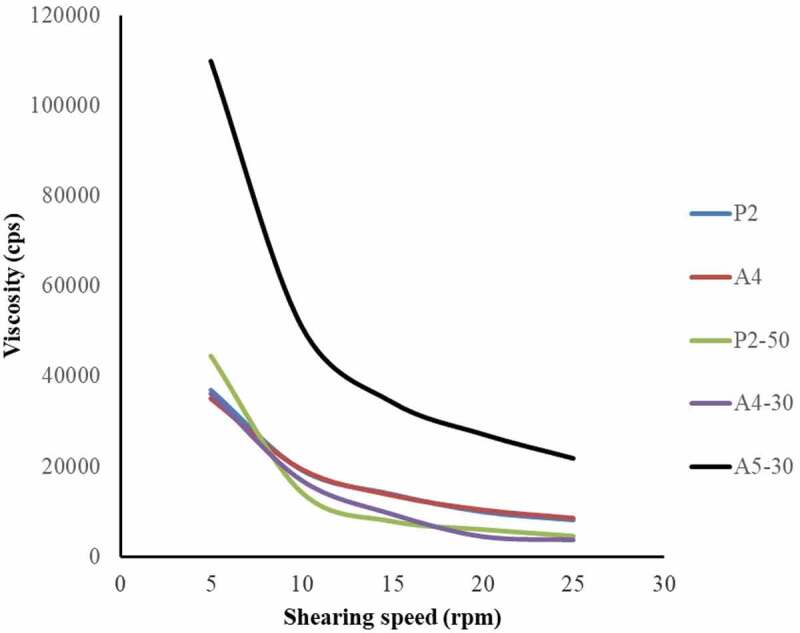
Rheological flow curve of CFS of *L. rhamnosus* SD11 sol–gel formulations P2-50, A4-30, A5-30 and blank sol–gel formulations P2 and A4

**Figure 7.**
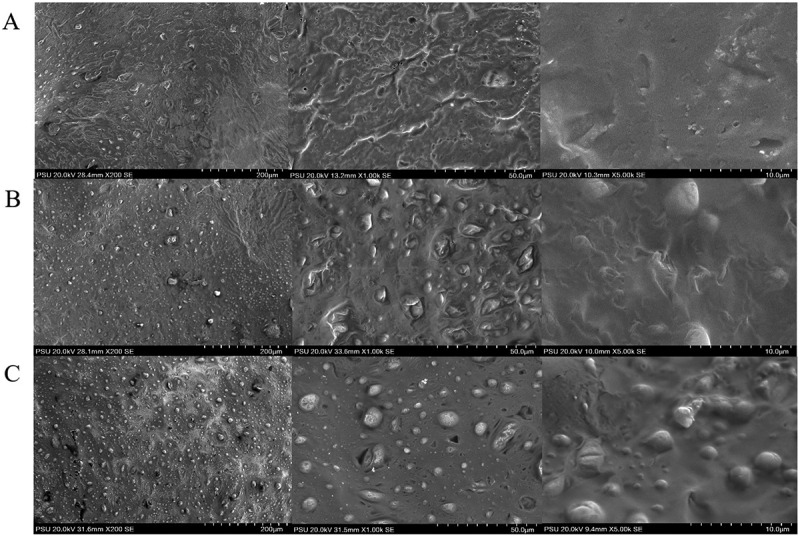
SEM photographs of CFS of *L. rhamnosus* SD11 sol–gel formulations P2-50 (**A**), A4-30 (**B**), A5-30 (**C**) with 200 X, 1,000 X and 5, 000 X magnification

The rheology profile of the blank copolymers (P2 and A4) and the CFS of *L. rhamnosus* SD11 sol–gel formulations (P2-50, A4-30 and A5-30) showed non-Newtonian viscosity (Figure 6). It was observed that the apparent viscosity decreased with an increase in stress, in all formulations. The mechanical properties of all five formulations (P2, A4, P2-50, A4-30 and A5-30) obtained using Texture Analyzer are displayed in Table 8. Blank sol–gel (P2) and CFS sol–gel formulations (P2-50) containing only poloxamer presented lower value of gel strength, rupture strength, brittleness and adhesiveness compared to other formulations. But it can be seen that in the sol-gel containing poloxamer (P2-50), the presence of CFS of *L. rhamnosus* SD11 increased values of rupture strength, distance of brittleness and adhesiveness, compared to the blank (P2) sol gel which had lower values. Sodium alginate which was added in the blank sol–gel formulation (A4) provided higher values of gel strength, rupture strength, brittleness and adhesiveness. The presence of CFS of *L. rhamnosus* SD11 in mixed polymer formulations of poloxamer and sodium alginate (A4-30 and A5-30) only increased the values of rupture strength and adhesiveness. The SEM photographs of the CFS of *L. rhamnosus* SD11 sol-gel formulations (P2-50, A4-30 and A5-30) are shown in Figure 7. The SEM photograph of P2-50 formulation showed a smoother surface area than the other two formulations (A4-30 and A5-30) which had rough and knobby surfaces. This could be due to the sodium alginate contained in these formulations.

**Table 8.** Mechanical properties of the selected gel formulations containing CFS of *L. rhamnosus* SD11 and blank gel formulations (37°C)

Formulations	Gel strength (g)	Rupture strength (g)	Brittleness (mm)	Adhesiveness (g.sec)
P2	5.49 ± 0.65	5.49 ± 0.65	3.00 ± 0.00	−22.29 ± 2.15
A4	9.08 ± 0.03	18.38 ± 0.43	10.00 ± 0.01	−43.26 ± 1.70
P2-50	5.06 ± 1.36	14.38 ± 2.58	10.00 ± 0.00	−26.3 ± 2.43
A4-30	10.42 ± 0.64	24.06 ± 0.18	10.00 ± 0.01	−58.76 ± 1.05
A5-30	10.52 ± 0.66	20.55 ± 0.45	10.00 ± 0.00	−65.49 ± 3.82

Adhesiveness is defined as the negative force area for the first compression cycle

**Figure 8.**
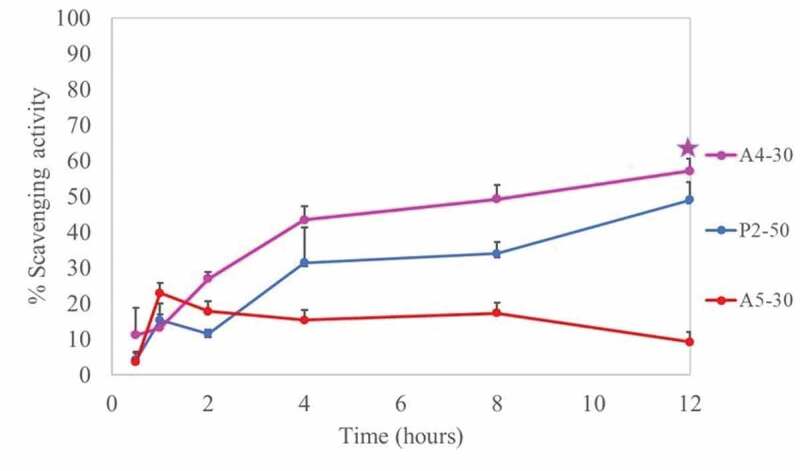
Release profile of formulations containing CFS of *L. rhamnosus* SD11. P2-50 was composed of poloxamer 407 (16% w/w) and CFS (16% w/w), A4-30 was composed of poloxamer 407 (15% w/w), sodium alginate (0.5% w/w) and CFS (10% w/w), A5-30 was composed of poloxamer 407 (16% w/w), sodium alginate (0.5% w/w) and CFS (10% w/w). All data are expressed as mean ± SD, n = 5. * is significant difference (*P*-value = 0.039) between A4-30 and A5-30

### Release profiles of sol-gel formulations containing *L.* *rhamnosus* SD11

The release profile was determined by the percentage of antioxidant activity at various time interval periods, and is shown in Figure 8. It was seen that formulation A4-30 had the highest release of CFS of *L. rhamnosus* SD11 because of its low viscosity (Table 6). All the formulations displayed a rapid release of active ingredient in the first h, after which a sustained release was seen for the remaining 11 h.

### Stability study

**Figure 9.**
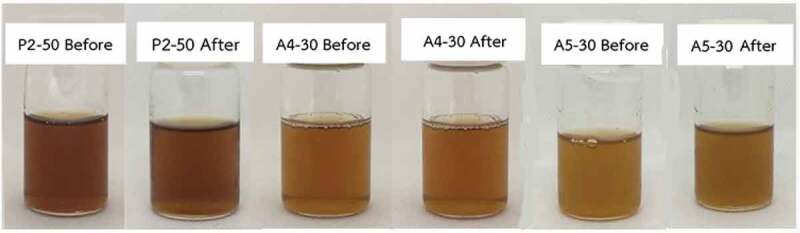
Appearance of formulations containing CFS of *L. rhamnosus* SD11 before and after freeze-thaw cycles

The stability of all three formulations containing CFS of *L. rhamnosus* SD11 (P2-50 and A4-30 and A5-30) were assessed by their appearance, pH and antioxidant activity, before and after freeze-thaw cycles. The appearance of all formulations is exhibited in Figure 9. pH results of all three formulations (P2-50 and A4-30 and A5-30) before and after the freeze-thaw cycles were 7.30 ± 0.02, 7.07 ± 0.02, 7.17 ± 0.03 and 7.20 ± 0.02, 6.84 ± 0.04, 7.01 ± 0.03, respectively. The results of antioxidant activity before and after freeze-thaw cycles were 67.79 ± 1.98, 61.15 ± 7.19, 69.52 ± 4.63 and 73.12 ± 4.49, 62.66 ± 5.28, 75.70 ± 0.52, respectively. There was no significant change in appearance, pH and antioxidant activity. All formulations were stable with unchanged physicochemical properties.

## Discussion

*S. mutans* and *S. aureus* are Gram-positive bacteria that can cause dental decay and oral infection respectively [20,21]. CFS of *L. rhamnosus* SD11 displayed good antimicrobial effect on Gram-positive bacteria according to a previous study [4] and it was also shown that, its antimicrobial activity against *S. mutans* was much better than that for *S. aureus*. The CFS of *Lactobacillus* consists of various bio-active compounds such as bacteriocin, proteins, short chain fatty acids, hydrogen peroxides and other cell wall fragments which contribute to antimicrobial activity. Therefore, the synergistic effect displayed by CFS of *L. rhamnosus* SD11 could be an outcome of summation of the activities of bacteriocins, organic acids, and hydrogen. In the antioxidant activity study, it was observed that the DPPH quenching capacity of the CFS strain increased with an increase in concentration. Previous studies have also reported that the antioxidant activity of cultured supernatant from lactic acid bacteria depended on the concentration [22,23]. In the DPPH scavenging assay, low EC_50_ value indicated potent antioxidant activity. Based on the EC_50_ values, CFS of *L. rhamnosus* SD11 possessed lower antioxidant capacity compared to that of ascorbic acid. However, this value of antioxidant activity could still be considered high enough. Damage caused by free radicals is a major contributor to various diseases and inflammation, and the presence of antioxidants can protect cells from this kind of damage. Therefore, antioxidant activity could play an important role in preventing diseases such as tooth decay and periodontitis and maintaining good oral health [24]. The pH of all formulations was adjusted to a suitable range of 6.2–7.6 [25] using triethanolamine, so that it remained in the pH range similar to that of human saliva. Comparison of gelation temperature values showed that when the concentration of poloxamer 407 increased, it resulted in a decrease of gelation temperature. This result corresponded to the study of Gelara et al. [26] which showed that the gelation temperature can be dependent on the molecular weight and concentration of polymers in a formulation. This phenomenon could be explained by the degree of swelling of the covalently linked networks in thermoresponsive polymers, where an increase in concentration could increase the degree of swelling, thereby reducing the temperature at which the gelation occurs [7]. The concentration of poloxamer 407 in the previous study was 5, 10, 15 and 20%, and the results showed that when the concentration was 15% w/w, the gelation temperature was 39°C, while a concentration of 20% w/w had a gelation temperature of 28°C [26]. In our study, the selected concentrations of poloxamer 407 were in the range of 15–20% (15, 16 and 17% w/w) because it was noted from the previous study that these concentrations could give a gelation temperature which was closer to the human body temperature (37°C). In our study, the formulations containing poloxamer 407 and xyloglucan showed a lower gelation temperature compared to the formulations containing poloxamer 407 only. This was in opposite to the results of Rangabhatla et al. [16] where the formulations containing poloxamer 407 and xyloglucan showed a higher gelation temperature compared to formulations containing poloxamer 407 only. This might be due to the difference in properties of xyloglucan used in both studies. The gelation temperature of A1 was over 40°C, which was why the formulation was still liquid at 37°C, and not a semisolid gel that could adhere in the cavity of the root canal. It could be concluded that addition of active ingredients and other compounds to the poloxamer 407 can increase or decrease the gelation temperature [8]. Furthermore, it was observed that increasing the concentration of polymers and active ingredient significantly increased all mechanical properties [27]. The flow behaviour of the blank and CFS of *L. rhamnosus* SD11 sol-gel formulation displayed shear thinning or pseudoplastic flow. Shearing can cause structural changes in a formulation, and once the structural change occurs, subsequent shearing can promptly decrease the apparent viscosity according to a previous study [27]. This was similar to the result we obtained as well, but this flow characteristic is not very important in our study as the sol-gel formulation will be used in dental caries. In this study, gelation temperature of the formulation was considered the most important, because the formulation is applied in the dental cavities using a syringe, and the teeth cavity structure have to hold the formulation inside firmly. Chewing force could create a shearing effect, but this shearing effect would be too small to have any inluence on the formulation. However, the formulation containing only poloxamer might be affected by the chewing force because of its low gel strength, brittleness and adhesiveness. From the release profile results, it was observed that the viscosity of the formulation could have a greater effect on release properties (P2-50 and A4-30), even more than the amount of active ingredient. However, when the viscosity of the formulations was of similar value, the formulation with the higher amount of active ingredient gave a higher release value (P2-50 and A5-30). In addition to the fact that poloxamer 407 is a weak mucoadhesive, it has poor mechanical properties and short residence time due to easy dissolution at the action site [28]. However, the release capacity is not only affected by the concentration gradient, but also by the viscosity, the polymer tortuous structure and characteristic of the polymer. The release characteristic might be a combination of all these factors or a single dominant factor depending on the formulation ingredients. It could also be observed in the release profile results that all the formulations displayed a rapid release of active ingredient in the first h, after which a sustained release was seen for the remaining 11 h. This effect could be explained by the structure of thermoresponsive copolymer and also the type of polymer solution used in our study because, poloxamer 407 has a lower critical solution temperature (LCST), which means that at low temperatures the polymer will remain a solution, and it changes to a hydrogel at higher temperatures. When the polymer solution transitions to gel, it causes a rapid decrease in volume of the gel resulting in rapid release of entrapped drug on the outer part of the hydrogel, followed by a sustained release of the drug entrapped inside the gel structure [7]. This phenomenon also explains the rapid release of the active ingredient in the first h of our release profile. Moreover, SEM photographs of A4-30 and A5-30 showed that the structure of the mixed polymer between poloxamer and sodium alginate was rough and knobby which might be due to the sodium alginate. Therefore, the formulation of A4-30 and A5-30 could penetrate into deeper areas when it was a solution, and made steric hindrance inside the root canal when it changed to gel. Formulation A5-30 had higher viscosity, higher adhesiveness and a lower release than A4-30, which is why A5-30 could not be a good candidate to create a formulation for dental prophylaxis.

The previous clinical studies of *L. rhamnosus* SD11 used two vehicles (milk powder and fermented milk for preventing dental carries [2,3,5]. Either milk powder or fermented milk, containing *L. rhamnosus* SD11 was able to have a similar effect on prevention of the caries risk. However, milk powder had an advantage over fermented milk because of longer shelf‐life, easier storage, and easier handling and transportation. Furthermore, CFS of *L. rhamnosus* SD11 was also studied for its potential of dental carries prophylaxis [2,4]. Thermoresponsive sol–gel formulation was chosen to incorporate CFS of *L. rhamnosus* SD11 because this type of formulation might be able to effectively deliver the active ingredient directly to the small holes in dental carries, in order to delay and prevent serious tooth infection. This type of formulation could also become an alternative pharmaceutical product for individuals who are allergic to dairy products. Poloxamer 407 is the main polymer in this formulation, which is safe because it is called an ‘inactive ingredient’ by the U.S. Food and Drug Administration (FDA), and used in many kinds of drug products such as oral solutions, suspensions, inhalation formulations, and intravenous, ophthalmic and topical formulations [8]. Preservatives are added to pharmaceutical products to prevent any kind of physical, chemical or biological changes. Therefore, a preservative was used in both blank and CFS of *L. rhamnosus* SD11 sol–gel formulations to prevent products from microbial contamination and to prolong their shelf life, especially since there was a high amount of water in these formulations. Moreover, methylparaben and propylparaben are still used in pharmaceutical products within the range of safe concentrations [29]. A minor disadvantage of this formulation is that it needs to be filled in a syringe for administration into the root canal. It might be uncomfortable for some people to use it themselves, which may need administration by a dentist.

## Conclusion

This study was successful in developing a thermoresponsive copolymer sol–gel formulation. The formulation that was most suitable (A4-30) contained 15% w/w of poloxamer 407 and 0.5% w/w of sodium alginate, and it could deliver 10% w/w of CFS of *L. rhamnosus* SD11. It had good physicochemical properties and stability, and gave a high release of active ingredient. The syringeability and injectability were also good with an appropriate gelation temperature, which is especially useful for root canal treatments. The antimicrobial and antioxidant activity were also high, which proves that the formulation would be effective in dental carries prophylaxis.
